# Pediatric pulmonary actinomycosis: A misdiagnosed case report

**DOI:** 10.3389/fped.2022.955554

**Published:** 2022-12-06

**Authors:** Zhao Yilin, Liu Xinyi, Sun Longwei, Zeng Hongwu

**Affiliations:** ^1^ Department of Radiology, Shenzhen Children's Hospital, Shenzhen, China; ^2^ Department of Radiology, Shenzhen Children's Hospital, China Medical University, Shenzhen, China; ^3^ Department of Radiology, Shenzhen Children's Hospital, Shantou University Medical College, Shenzhen, China

**Keywords:** actinomycosis, lung, child, disease attributes, diagnosis

## Abstract

**Background:**

Pulmonary actinomycosis is an uncommon kind of bacterial illness caused by actinomycetes, involving the chest wall in extraordinarily rare cases. Due to non-specific clinical signs and perplexing radiological characteristics, this kind of pulmonary actinomycosis is frequently misinterpreted as a malignant tumor or lung abscess.

**Case presentation:**

An 11-year-old child presented with a palpable lump on his left chest and periodic chest discomfort. An irregular soft-tissue mass in the left upper zone with bony destruction was first identified as a malignant small round cell tumor (MSRCT) known as an Askin tumor on post-contrast CT. However, pathological biopsy of the pulmonary lesion through the chest wall revealed actinomycosis.

**Conclusion:**

Pulmonary actinomycosis is an uncommon bacterial illness that has a variety of clinical manifestations, particularly in young patients. A chest lump with nearby “lace-like” rib bone destruction was the distinguishing characteristic of our case. For appropriate treatment and diagnosis, infection with actinomycosis should be considered when observing a similar chest lump. Pathological biopsy, as a valuable diagnostic tool, can help to distinguish between infectious diseases and thoracic tumors. The pathological manifestations of actinomycosis are characterized by inflammatory lesions that range from purulent to granuloma-like inflammatory processes, and second-generation sequencing of alveolar lavage fluid can help to confirm pathogens.

## Introduction

Actinomycosis is a rare and chronic suppurative granulomatous infection mainly caused by Actinomyces israelii, first reported in 1857 (1). It occurs most commonly in the face and neck (50%–60%), the abdominal and pelvic cavity (20%), and the chest (15%), while it is extremely rare in the lungs (2). In terms of age distribution, it is most commonly seen in middle-aged adults and very rarely in children; it affects two to four times more males than females (3). Lacking specific symptoms, pulmonary actinomycosis in children is usually indistinguishable from pulmonary tuberculosis and lung abscess when a chest radiograph or CT reveals a single mass in the lung. When the ribs, pleura, and chest wall are involved, it may mimic thoracic malignancy in radiographic findings. Therefore, we report a case of caries-related pulmonary actinomycosis in an 11-year-old boy, misdiagnosed as a malignant small round cell tumor (MSRCT) identified as an Askin tumor of the chest wall.

## Case presentation

An 11-year-old boy presented with occasional chest pain for 2 weeks. A palpable mass was initially found on his left chest. He denied having a cough, chest stuffiness, polypnea, or weight loss. Dental caries was observed. On physical examination, an 8 cm × 7 cm palpable mass was found on the left upper chest wall; there was tenderness of the mass but no swelling; and skin temperature was normal. Retraction to a slight extent and decreased breath sound were observed over the left lung field.

**Figure 1:**
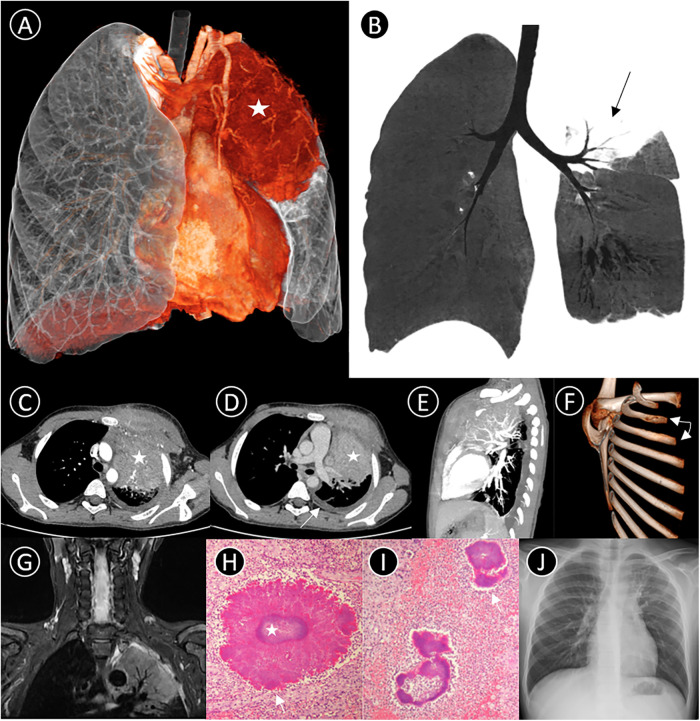
(**A**) 3D-VR image shows a mass lesion (star) in the left upper lobe; (**B**) coronal Minimum intensity projection (MinIP) indicates some air bronchus signs (black arrow); (**C–E**) axial and lateral contrast-enhanced CT thorax scan shows a large soft tissue density mass (star) in the left upper region, measuring 6.5 cm × 8.5 cm × 7 cm in size, with moderate heterogeneous enhancement. The mass invades the adjacent rib (triangle, left 2nd and 3rd rib) and chest wall soft tissues, and an associated pleural effusion is observed medial to the left crus of the diaphragm (white arrow); (**F**) 3D-VR image demonstrates bony destruction with periosteal reaction in left 2nd and 3rd ribs (white arrow); (**G**) T2-weighted image demonstrates the left upper lobe presenting as hyperintensity with air bronchogram sign, involving the pleura and muscles of the anterior chest wall; (**H,I**) Pathological findings suggest septic inflammatory changes in the lesion tissue, and show actinomycotic granules with a basophilic center containing bacteria colonies (star) with eosinophilic periphery composed of Splendore–Hoeppli material (white arrow) (PAS stain, PAS-D stain, 1,000×); (**J**) Following antibiotic treatment for 2 months, chest radiograph shows most of the lesion having been absorbed.

Laboratory tests showed neutrophilic leukocytosis of 13.27 × 109/L, hemoglobin of 105 g/L, platelets of 524 × 109/L, C-reactive protein level of 44.59 mg/L, 70.9% lymphocytes, and 0.2% eosinophils, and a Mantoux skin test was negative. The patient underwent a detailed series of radiological examinations to visualize the entire landscape of the lesion. 3D-VR imaging and MinIP showed the mass and its internal structures (Figures 1A,B).

Post-contrast CT and MRI revealed an irregular soft-tissue mass in the left upper zone. Several small arteries could be seen emerging from the left pulmonary artery in the lesion. There were some bony invasions of the left 2nd and 3rd ribs and an associated left pleural effusion (Figures 1C,G). Because of its radiological characteristics, a type of malignant small round cell tumor known as an Askin tumor was initially considered. Subsequently, bronchoscopy demonstrated endobronchial inflammation with a little yellow and white secretion before antibiotic treatment. However, pathological biopsy *via* the chest wall suggested purulent inflammation with scattered bacterial clusters, which morphologically suggested Actinomyces species (Figures 1H,I). Further to this discovery, Actinomyces odontolyticus bacteremia, Actinobacillus actinomycetemcomitans, and Fusobacterium nucleatum were detected in bronchoalveolar lavage fluid next-generation sequencing. High-throughput DNA sequencing eventually proved infection of Actinobacillus actinomycetemcomitans (bacteria), Haemophilus influenza (bacteria), and Alterniospora (fungus).

The patient was treated with an intravenous infusion of cefoperazone sodium and sulbactam sodium for 9 days while the etiology was unknown. After confirmation of actinomycosis, treatment was switched to penicillin combined with piperacillin/tazobactam and included a one-month regime of gargling using compound chlorhexidine gargle. After discharge, the patient changed to oral amoxicillin for 9 weeks. At 2-month outpatient follow-up, the patient had completely recovered, with no symptoms remaining, and chest radiograph showed that most of the lesion was absorbed (Figure 1J).

## Discussion

Pulmonary actinomycosis accounts for about 15% of all actinomycosis. However, chest wall involvement is extremely rare. Due to a lack of typical clinical appearance and confusing radiological features, this rare disease usually masquerades as tuberculosis, chronic suppurative pulmonary disease, or malignancy. Early and accurate diagnosis can avoid unnecessary surgical intervention (3). Actinomycete is a gram-positive, non-spore-forming, and anaerobic prokaryotic bacterium, which forms part of the normal bacterial flora in the oropharynx, gastrointestinal tract, and female genital tract. When the mucosal barrier is destroyed, actinomycetes can invade the tissues and organs, and thereby cause endogenous infection (4). Pulmonary actinomycosis usually results from aspiration of oropharyngeal or gastrointestinal secretions, while pediatric pulmonary actinomycosis can also be spread through the blood. In children, dental caries, trauma, reduction of immunity, poor control of blood glucose, and foreign body aspiration are the major potential causes of actinomycosis (5). However, previous studies have reported that pulmonary actinomycosis can also occur in adults and children without immunodeficiency or any underlying disease (6).

The clinical manifestations of pediatric pulmonary actinomycosis include cough, expectoration, chest pain, fever, and a lack of specificity of presentation. According to a recent report, the symptoms of pulmonary actinomycosis in children are similar to those of a patient with a tumor (weight loss, etc.) or tuberculosis (low fever and night sweats). Obvious weight loss, feeling unwell, and high fever could warn of the possible spread of pulmonary actinomycosis (7). Laboratory biochemical examination may display an abnormal number of leukocytes, manifesting primarily in the form of an increased number of neutrophils with or without increasing C-reactive protein. Due to analogous test results in other common inflammations, routine blood examination is less competitive in the diagnosis of pulmonary actinomycosis.

Pulmonary actinomycosis is associated with diverse radiology findings at different periods. In the early stage, irregular peripheral pulmonary nodules near the pleura with a hazy border similar to a halo sign may be found on CT. Some radiologists consider the halo sign to be associated with local invasion or pulmonary hemorrhage (8). In contrast, in the middle stage, it can progress as a pulmonary mass with adjacent lung consolidation and internal patchy areas of hypodensity indicating colliquative necrosis. The typical imaging finding at this stage is suspended air bubbles with no air-fluid levels due to a lack of mobile liquid(9). CT can reveal the pathology behind the radiology findings; within the mass, low-density areas are composed of necrosis, actinomycete, and sulfur granules, while areas of air density are micro-abscesses and residual cystic bronchiectasis (10). Unlike a lung abscess, pulmonary actinomycosis may sometimes spread across pulmonary segments or even lobes, which could be helpful for differential diagnosis (11). Invasion of adjacent ribs and the resulting bone destruction and periosteal reaction is an extremely important phenomenon for diagnosis (12). When pleural and chest-wall effusion occurs, pulmonary actinomycosis presents as a pulmonary mass combined with a chest wall mass, mimicking cancer derived from the chest wall. In a contrast-enhanced CT scan, the solid part of the lesion shows obvious and progressive enhancement, and the time–density curve is of a fast–plateau–slow type, in line with that of granulomatous inflammation. Second-generation sequencing of alveolar lavage fluid can also function as an alternative diagnostic instrument.

Obtaining stable positive pathological specimens is critical to confirm the diagnosis of pulmonary actinomycosis. Because conventional alveolar lavage fluid is easily exposed to air, the strictly anaerobic nature of actinomycetes makes it difficult to cultivate actinomycete clusters. The pathological biopsy is the gold standard for identification of pulmonary actinomycosis without typical clinical characteristics. Bronchoscopy biopsy or CT-guided percutaneous aspiration pathology biopsy is the most commonly used test and has high efficiency (13). Sulfur particles detected in pus, sinus exudate, or lesion tissues are highly suggestive of pulmonary actinomycosis. Nevertheless, such particles are not the most crucial diagnostic evidence because they can also appear in other infectious diseases, such as nocardiosis or fungal heart disease (14). Sulfur particles, composed of actinomycete and surrounding tissue fragments, appear as round, oval, or horseshoe-shaped basophilic masses surrounded by radial eosinophilic rods in hematoxylin–eosin staining. With the development of molecular technologies, more advanced and accurate detection methods have sprung up, such as 16S rRNA and whole-genome sequencing. Due to the high prices and limitations of sophisticated detection instruments, however, these advanced methods may not be employed routinely in most laboratories (4).

Although there is an extensive literature on actinomycosis therapy, most of the information is obtained from clinical case reports, and few publications focus on pediatric actinomycosis therapy. Antibacterial drugs for actinomycosis were first applied in 1938, in a case in which sulfonamides successfully cured an 11-year-old boy of his abdominal actinomycosis (15). So far, actinobacteria remain highly sensitive to *β*-lactam antibiotics, penicillin G, and amoxicillin. A long course of parenteral penicillin in large doses is a recognized recommendation in the field of therapy for actinomycosis, and ampicillin and ceftriaxone can be used as alternatives (16). The critical treatment strategy is the long-term use of antibiotics (usually for 6–12 months). The antibiotics of choice are penicillin G and amoxicillin. The most important preventive factor for children is oral hygiene (17). At present, there have been very few cases of pulmonary actinomycosis treated by surgery in children. Prompt surgery and long-term postoperative antibiotic treatment are both needed when drug treatment is not effective or severe complications occur, such as chest wall abscess, empyema, or chest fistula (18).

## Conclusion

Pediatric pulmonary actinomycosis is an extremely rare bacterial infection caused by a group of bacteria known as actinomycetes, usually misdiagnosed as pulmonary tuberculosis, lung abscess, or malignant tumor due to its lack of specific clinical symptoms or imaging features. Pulmonary actinomycosis is a granulomatous inflammation characterized by multiple abscesses, pus containing sulfurous granules, sinuses, and fistulas. Through production of lytic enzymes such as proteases, this form of actinomycosis is prone to spreading and infecting surrounding tissues, and hence appears to have a poorer prognosis than cervicofacial or abdominal actinomycosis. Suspended air bubbles, distribution across fissures, and lacy periosteal reaction may hold particular value in CT diagnosis of pulmonary actinomycosis, but go largely unacknowledged owing to the rarity of the disease.

Bronchoscopy and CT-guided percutaneous aspiration pathology biopsy can help to differentiate the diagnosis from tuberculosis, intratubular tumors, and foreign bodies. Overall, accurate diagnosis in the early stages and appropriate treatment can lead to a good prognosis.

## Data Availability

The original contributions presented in the study are included in the article/Supplementary Material, further inquiries can be directed to the corresponding author/s.
